# The good, the bad, and the opportunities of the complement system in neurodegenerative disease

**DOI:** 10.1186/s12974-020-02024-8

**Published:** 2020-11-25

**Authors:** Nicole D. Schartz, Andrea J. Tenner

**Affiliations:** 1 Department of Molecular Biology and Biochemistry, University of California Irvine, 3205 McGaugh Hall, Irvine, CA 92697 USA; 2 Department of Neurobiology and Behavior, University of California Irvine, 3205 McGaugh Hall, Irvine, CA 92697 USA; 3 Department of Pathology and Laboratory Medicine, University of California Irvine, 3205 McGaugh Hall, Irvine, CA 92697 USA

**Keywords:** Complement, Neurodegeneration, Neuroprotection, Alzheimer’s disease, Toll-like receptors, Microglia, Multiple sclerosis, Epilepsy, Traumatic brain injury, Stroke

## Abstract

The complement cascade is a critical effector mechanism of the innate immune system that contributes to the rapid clearance of pathogens and dead or dying cells, as well as contributing to the extent and limit of the inflammatory immune response. In addition, some of the early components of this cascade have been clearly shown to play a beneficial role in synapse elimination during the development of the nervous system, although excessive complement-mediated synaptic pruning in the adult or injured brain may be detrimental in multiple neurogenerative disorders. While many of these later studies have been in mouse models, observations consistent with this notion have been reported in human postmortem examination of brain tissue. Increasing awareness of distinct roles of C1q, the initial recognition component of the classical complement pathway, that are independent of the rest of the complement cascade, as well as the relationship with other signaling pathways of inflammation (in the periphery as well as the central nervous system), highlights the need for a thorough understanding of these molecular entities and pathways to facilitate successful therapeutic design, including target identification, disease stage for treatment, and delivery in specific neurologic disorders. Here, we review the evidence for both beneficial and detrimental effects of complement components and activation products in multiple neurodegenerative disorders. Evidence for requisite co-factors for the diverse consequences are reviewed, as well as the recent studies that support the possibility of successful pharmacological approaches to suppress excessive and detrimental complement-mediated chronic inflammation, while preserving beneficial effects of complement components, to slow the progression of neurodegenerative disease.

## Complement

### Complement cascade

Over 40 proteins are involved in the complement system, a part of the innate immune response which is critical for quickly recognizing and clearing pathogens, apoptotic cells, and cellular debris prior to generation of the adaptive immune system pathogen-specific response or cascading tissue-damaging inflammation. The system is also a dominant participant in antibody-mediated pathogen killing and clearance and contributes to directing the type and extent of the adaptive response (reviewed in [1]). Perhaps due to the ancient evolutionary origins of the pathway, it may not be surprising that in the past 15 years, multiple noncanonical functions of complement have been uncovered (reviewed in [2]).

**Fig. 1:**
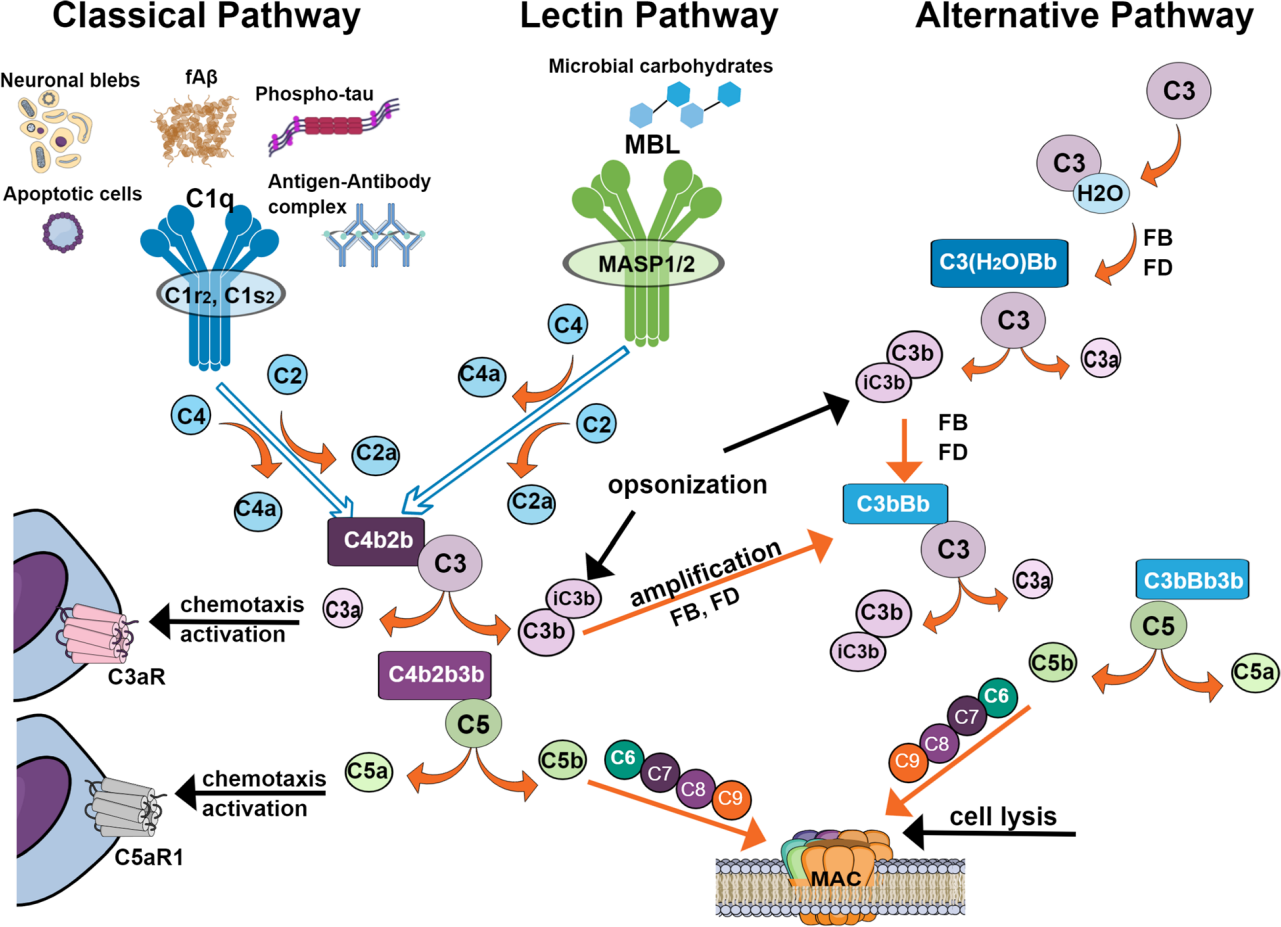
Activation pathways of the complement system. The complement system is activated by the classical, lectin, or alternative pathways. The classical pathway is activated when the C1 complex composed of C1q, C1r_2_, and C1s_2_, binds to apoptotic cells, neuronal blebs, fibrillar amyloid beta (fAβ), hyperphosphorylated tau, or antigen-antibody complexes via C1q. The lectin pathway is activated when mannan-binding lectin (MBL) in complex with MASP1/2 binds to microbial carbohydrates. Both pathways create the C3 convertase by cleaving C4 and C2 to form C4b2b. C3 is cleaved to form C3a, which promotes chemotaxis and activation of microglia via C3aR, and C3b can be cleaved to iC3b (by co-factors and factor I, not shown) to promote opsonization, or bind to C4b2b to form the C5 convertase (C4b2b3b). C5 is cleaved to form C5a, a potent inflammatory effector that acts through C5aR1 to promote chemotaxis and glial activation, and C5b, which binds to C6, C7, C8, and C9 to form the membrane attack complex (MAC) to permeate cell membrane and promote lysis. The alternative pathway can be activated when spontaneous hydrolysis of C3 to C3-H_2_O enables factors B and D to generate the C3 convertase (C3(H_2_O)Bb), which then cleaves other C3 molecules to C3b. The alternative pathway also forms an amplification loop for the other complement pathways, when factor B binds to C3b and is cleaved by factor D to form C3bBb which continues to cleave C3 to enhance the effect of activation. This also enables the C3bBb3b, alternative pathway C5 convertase, to cleave C5, releasing C5a and leading to the formation of the MAC

Complement is activated by three different recognition pathways (classical, alternative, and lectin), all of which lead to sequential enzyme activation, protein cleavage, and induced function-enabling protein conformational changes (Fig. 1). The classical complement pathway is activated when the C1 complex (C1q, C1r_2_, and C1s_2_) binds, via the C1q subcomponent, to the Fc domain of antibody in immune complexes or to nonimmunoglobulin activators such as apoptotic cells, or other polyanionic substances including fibrillar Aß [3] and hyperphosphorylated tau ( [4] and reviewed in [5]). This binding induces the autoactivation of the proenzyme C1r which then cleaves and activates C1s, another serine protease. Activated C1s cleaves component C4, generating C4a and C4b. C4b covalently attaches to the activator surface and recruits C2. C1s then cleaves C2 into C2a and C2b. C4b and C2b form the C3 convertase (C4b2b), which can sequentially cleave multiple C3 proteins into C3a and C3b. C3b also covalently attaches to the surface, providing the opsonic signal to phagocytes for ingestion (and subsequent killing or degradation). Some of the cleaved C3b remains associated with the C4b2b forming C4b2b3b, the classical pathway C5 convertase. The C5 convertase then cleaves C5 into C5a and C5b. C5b initiates the formation of the pore-forming C5b, C6, C7, C8, and C9 complex (C5b-9). The lectin pathway is quite similar to the classical pathway except that the recognition components, mannan-binding lectin (MBL), ficolins and collectins, and the MBL-associated serine proteases, MASP1/2, are activated by interaction with specific carbohydrate structures (not normally present in the host). Once activated, MASP1/2 cleaves C4 and C2 thereby forming the C3 cleaving enzyme (C4b2b) and enabling subsequent steps in the cascade. The alternative pathway of activation is the result of the direct attachment of cleaved C3b to a permissive/acceptor surface, which is often a pathogen but can also be other surfaces, ultimately resulting in the generation of the C3 cleaving enzyme complex C3bBb and the alternative C5 cleaving enzyme C3bBb3b [6] (Fig. 1).

Regardless of the activation mechanism, the same three major effector functions result: (1) opsonization, via the deposition of the activation dependent cleavage fragments C3b/iC3b (and to a lesser extend C4b and C1q) which tag pathogens for more efficient clearance by phagocytes, (2) leukocyte/microglia recruitment to the site of injury via production of chemotactic peptides, C3a and C5a, and (3) targeted death of pathogens due to the creation of a membranolytic pore in pathogen cell membranes initiated by C5b-9 (also known as the membrane attack complex, MAC). The generation of the diffusible chemotactic peptides, C3a and C5a, can also lead to the alteration of immune (and other) cell functional activities such as production of reactive oxidation species and secretion of pro-inflammatory cytokines that have evolved to aid in the efficient clearance of pathogens (reviewed in [2]). However, harmful inappropriate or excessive complement activation can lead to tissue injury with serious consequences including death. This is normally avoided by a number of circulating (C1 inhibitor, factor H, C4b-binding protein) and membrane-associated (CD46, CD55, CR1, CD59) complement regulators [6]. More recently, novel complement control proteins are being discovered, often with tissue-specific expression ([7] and reviewed in [8]), although the domain protein structures have similar features [9]. The impact that the complement system has on human health and disease has become even more evident with the recognition that combinations of specific complement component genetic polymorphisms, collectively referred to as a “complotype” [10] confer differential sensitivity to infection and autoimmunity. The complotype in the brain (distinct combinations of more or less robust activators and more or less robust inhibitors) may similarly influence susceptibility to various neurological disorders. In addition, the identification and ongoing characterization of intracellular complement activities, the “complosome,” has far-reaching consequences on cellular metabolism and on both innate and adaptive immune responses (reviewed in [11, 12]).

### Synthesis of complement proteins

The liver was originally thought to be the dominant site of complement protein production (reviewed in [13]). However, it became apparent that various complement components can be differentially induced in a variety of cell types [11, 14] and in the brain [15]. Importantly for our discussion here, complement components can be induced in CNS resident neurons, astrocytes, oligodendrocytes, cerebrovascular smooth muscle cells and microglia during development or induced by injury or aging (Table 1) as reviewed below, and more recently confirmed by single nucleus RNA-Seq [18]. While not constitutively produced in neurons, all complement proteins can be induced in neurons under a variety of conditions [37]. For example, C1q is clearly induced in neurons during development by astrocyte-secreted TGFß [38, 39]. With exceptions mentioned below, in both mouse and human, C1q is predominantly expressed in microglia in the adult brain [16, 40] and upregulated with age [41], injury and interferon γ, compatible with PU.1 and IRF8 elements in the promoter that controls the synchronous transcription of all 3 C1q subunits (A, C and B) [42]. C4 was shown to be induced in oligodendrocytes in mouse models of AD by immunohistochemistry [43] and more recently via single nucleus RNA-Seq [18]. Transcriptomics has also shown age-related increases in C4 in murine astrocytes in all brain regions tested [44, 45], which is further induced by lipopolysaccharide (LPS) (A1 astrocytes) [28], and in disease-associated astrocytes (DAA) [46]. Interestingly, C4 was predominantly expressed in astrocytes in AD brain versus in the oligodendrocytes seen in murine 5xFAD models [18]. C3 as well as C1r, C1s, and C2 can also be notably induced in murine brain with aging and in “A1” astrocytes [17, 28, 43, 44, 46]. Lian and colleagues showed that TNFα induced NfκB and C3 in astrocytes, consistent with putative κB-binding sites in the C3 promoter [47]. Thus, while C1q is secreted at the earliest times of injury, other glia may subsequently be induced to provide the other components needed for complement activation—for both synapse pruning and complement-dependent neurotoxicity as elaborated below. Interestingly, the terminal complement components are less often detected, and at lower levels, than the earlier components [48], supporting a transcriptional level of regulation of the functions of this potent cascade in the brain. An exception to this appears to be mRNA for the C8γ chain, which is present in CNS in the absence of C8a and C8b chains ( [49] and unpublished data), suggesting it may have a functional role independent of the formation of the complement membranolytic pore. Importantly, all of these studies demonstrate that, while in many neurodegenerative disorders the blood-brain barrier may be transiently compromised allowing entry of constitutively present blood complement proteins, many complement proteins are present in both human and mouse injured/aging brain even with an intact blood-brain barrier.

**Table 1:** CNS cell-specific upregulation of complement component genes in humans and animal models of neurodegenerative diseases

	AD	ALS	Stroke	Epilepsy	TBI	MS
Microglia	C1q, C3, CR4, C3aR [16, 17]	CR4, C1q [22]	C1q [23]	ND	CR4, C1inh, C1s, C3, C4 [24]	C3, CD40, CR4, CR3 [25, 26]
Astrocytes	C4, C3, C1inh, clusterin [17, 21, 27]	ND	C4, C1rb, C1ra, C1s, C1inh [28]	ND	C1q [29]	C3, C1inh, CR4, C3, C4, C1q [30]
Neurons	*C1q*, *C2*, *C3*, *C4*, *C5*, *C6*, *C7*, *C8*, *C9* [31]	*C1q*, *C4*, *C3* [32], C1q, C5aR1; CD55 is downregulated [33, 34]	C5, C5aR [35, 36]	ND	ND	ND
Oligodendrocytes	C4 [18]	ND	ND	ND	ND	ND

*Italics*=human tissue, *ND *not determined

### Homeostatic and regulatory functions of C1q independent of complement activation

C1q can be synthesized in the absence of the C1 serine proteases C1r and C1s in peripheral myeloid cells [50]. In the CNS, synthesis of C1q has also been shown to be upregulated as an early response to injury in many cases (reviewed in [51]) also without the concomitant synthesis of the classical pathway serine proteases suggesting C1-independent C1q-mediated functions (reviewed in [52, 53]). Interaction of C1q but not C1, with myeloid cells, including microglia, has been shown to enhance clearance of apoptotic cells and neuronal blebs and suppress proinflammatory cytokine production [54–56]. Furthermore, C1q has a direct protective effect on primary cultured rodent neurons under nutrient stress or amyloid-induced toxicity, again without the presence or activation of any downstream components of the complement cascade [57–59]. Whether these consequences are relevant to the synapse stabilization roles of the C1q-like family of proteins under active investigation [60] and reviewed in [61] remains to be seen. In any event, identification of the neuroprotective pathways triggered by C1q should provide novel candidates for therapeutic targeting. The current availability of a mouse with a floxxed C1qa gene that can induce C1q deletion in adult brain [16] should enable the determination of the extent to which these activities influence pathology and/or dysfunction in many disorders, without the caveat of absence of the protein (C1q) during development and/or lifelong deficiency.

### C1 through C3 function in synapse pruning during development, in the adult and in neurologic diseases

In a series of innovative experiments, C1q along with C3 (and necessarily C1r, C1s, C4, and C2) was found to be involved in beneficial synapse pruning (refinement) during retinal development and functional maturation of interneurons [39, 62], and more recently in the process of forgetting [63]. This pruning involves microglial ingestion via the complement receptor, CR3 [64], although there is evidence that astrocytes may play a role in this activity as well [65]. However, several groups have provided evidence for excessive complement-mediated synapse pruning in animal models of aging, AD models, and other disorders that display cognitive or behavior impairments [66–69] and more recently in motor neuron disease [70]. Using high- and super-resolution microscopy, C1q can be observed colocalized with either pre-synaptic or post-synaptic markers [39, 67, 71]. Accumulation of C1q-tagged synapses is also reported in human diseases, such as tauopathies, Alzheimer’s disease (AD), West Nile virus-induced cognitive loss, and more [17, 68, 69]. In human iPSC-derived neurons, loss of surface sialic acid permitted C1q binding and subsequent removal of neurites via CR3 (iC3b/C3b receptor) expressing macrophages [72, 73]. Proteomic approaches show C1q-tagged synaptosome preparations to contain higher levels of cleaved caspase 3 and annexin 5, both markers of apoptosis, than C1q-negative synaptosomes [74], suggesting similar mechanisms between synapse pruning and clearance of apoptotic cells. While the initial site/molecular component to which C1q binds at the synapse has not been definitively assigned, consistent with similarities to ingestion of apoptotic cells, dissipated CD47 [75], surface-exposed phosphatidylserine or annexin V [74], decreased mitochondrial functions, and altered synaptic septin organization [76] have been associated with enhanced C1q binding and synapse engulfment in developmental or injury models. Interestingly, while both C1q and C3 fragments are seen in postmortem human brain tissue from multiple sclerosis (MS) patients [77], recent studies have reported that C3, but not C1q, is required for loss of retinogeniculate synapses and visual acuity in multiple MS-relevant animal models [78], suggesting that complement activation by classical pathway (involving C1) is not the only means of tagging synapses for engulfment (see below). However, importantly, it appears that loss of synapses may be a separate feature of neurodegenerative diseases; as demonstrated in a model of MS suppression of C3 cleavage resulted in inhibition of synaptic engulfment by microglia and protection of visual acuity, while not affecting gliosis, demyelination or other measures of disease progression [78]. Additional mechanistic studies are necessary to clarify the parameters involved in beneficial and detrimental complement-mediated synaptic pruning.

### C5a and C3a as mediators of neuroinflammation

Evidence from multiple systems suggests that inflammation due to the complement system is induced by the activation-induced cleavage products, C5a and C3a, although the physiologic effects are highly context specific. C5a engagement of C5aR1 (also known as CD88), a G-protein coupled 7-transmembrane receptor, leads to induction of chemotaxis, degranulation, cytokine production, and other often cell type-specific responses (reviewed by [1]). Multiple publications report conflicting expression of C5aR1 particularly in the brain. Some of the confusion is derived from the cross-reactivities of monoclonal antibodies particularly in immunohistochemical detection [79] and from the use of immortalized cell lines which may or may not mirror the in vivo expression of the receptor. However, the potential for brain region-specific differential cell expression, induction of expression of these receptors in various injury or disease states, and the presence of context-specific co-receptors/factors ( [80] and reviewed in [81]) provide valid layers of functional complexity, yet also opportunities for specificity in therapeutic targeting (reviewed in [1, 82]). In addition, there is a second confounding receptor for C5a, C5aR2, originally designated C5L2, that acts as a counterbalancing receptor for C5a [83, 84]. The use of specific inhibitors of C5aR1 and the availability of C5aR1 knockout mice (that can be used as negative controls for immunoreactivity and functional responses to C5a) are leading to reliable and corroborating results [36, 85, 86]. In addition, with the rapidly evolving single-cell RNA-Seq and RNAScope technology, these apparent conflicting studies are and will continue to be resolved.

C3a, the cleaved activation fragment that results from activation of all three complement activation pathways, signals through the G-protein coupled 7 transmembrane C3aR. It is becoming apparent that the outcomes of this C3a signaling are diverse and context specific [87]. In addition, emerging data indicate that other peptides induced in injured brain signal through C3aR [88] as will be discussed below.

Pattern recognition receptors, such as Toll-like receptors (TLRs), are another important aspect of the innate immune system that can synergize with or antagonize the complement system to tightly regulate the response to pathogens and misfolded proteins (including Aβ or tau, see below). In the periphery, C5aR1 was found to synergize with TLR2 and TLR4 and to enhance pro-inflammatory cytokine responses (TNF-α and IL-1β) when using in vivo mouse models, mouse macrophages, and human monocytes (reviewed in [89]). These data combined with a large body of previous investigations of non-CNS C5aR1 intracellular signaling (reviewed in [90]) indicate that the C5a-C5aR1 axis produces a pro-inflammatory environment by acting on MAPKs, leading to generation of inflammatory cytokines some of which are known to be expressed in inflamed brain. Specific antagonists of C5aR1 have been shown to inhibit peripheral acute C5a-induced inflammatory disorders as well as chronic debilitating disorders in animal models (Table 2) [123, 124]). As discussed below, there is promising evidence that blocking C5aR1 signaling may dampen the inflammatory response and decrease neuronal injury, thereby slowing loss of neuronal function, including cognition [5].

**Table 2:** Therapeutic potential of inhibition/deletion of Complement components in models of neurodegenerative diseases

	Classical	Lectin	Alternative	C3	Terminal (C5a, C5b-9)
AD	C1q KO decreases amyloid plaques, gliosis, and hippocampal neuronal loss in Tg2576 model [91]			C3 KO attenuates synaptic loss, gliosis, cognitive decline in APP/PS1 model [92]	C5aR1 KO attenuates cognitive decline, promotes microglial clearance, protects neuronal integrity in Arctic model [93]. PMX205 reduces plaque load, cognitive loss in Tg2576/3xTg [94]
ALS	Deletion of C1q or C4 does not alter disease onset or severity suggesting classical pathway is not essential [82, 95]				PMX205, C5aR1 KO, C6 ASO in SOD^G93A^ mice delays progression, extends survival, improves motor function [82, 96, 97, 98, 99]
Stroke	C1q KO [100], C1qsiRNA [101], C1inh [102] reduces infarct volume, promotes neurogenesis, protects BBB	MBL KO mice have improved neurological scores, reduced infarct volume, reduced C4 deposition [103, 104]	FB KO, CR2-fH treatment improved neurological scores, reduced infarct volume after MCAO [105]	C3 KO [106], B4Crry treatment [107] reduces infarct volume, inflammation, improves neurological function	C6 KO is not protective in MCAO and CD59a KO does not reduce damage, suggesting terminal complement pathway is not essential for MCAO injury [105]
Epilepsy	C1q KO increases hyperexcitability and risk of spontaneous seizures [62, 108]			Intravenous immunoglobulins (IVIg) blocks C3 & reduces spontaneous seizures after SE [109]	C5 KO mice have fewer seizures [110], PMX53 reduces seizure length, probability of subsequent seizure in kainite model [111, 112]
TBI	C1inh, anti-C1q Ab [113], C4KO [114] reduces lesion size, improves cognitive and motor outcomes		FB KO [115], CR2fH [116] reduces glial scarring, neuronal death, lesion size, motor and cognitive deficits	C3 KO [113], CR2Crry [116] improves motor and cognitive function, reduces scarring long term.	CR2-CD59 [117], C6 ASO, & OmCl [118] reduce neurological deficits, inflammation, neuronal damage short term only [116]
MS (EAE)	C4 KO has no effect, suggesting classical pathway is not essential [119]		FB KO or FB antibody results in delayed onset and reduced severity of symptoms [120]	sCR1 & CVF attenuate demyelination, disease severity [121, 122]	

## Alzheimer’s disease

Currently, there are 5.8 million people in the US with AD whose care in 2020 is estimated to cost $305 billion [125], and greater than 50 million suffering from dementia worldwide. Unfortunately, there are no currently approved treatments for the synaptic loss or neuronal death and dysfunction, which is the cause of cognitive loss in AD and the most impactful clinical presentation of this disease. Initial observations in early-onset (< 60 years of age) AD patients revealed that many possessed inherited mutations in the amyloid precursor protein (APP) gene itself or in the genes coding for presenilins 1 or 2 (PS1, PS2), the enzymes that cleave APP to generate the amyloid ß peptide. This, and the fact that plaques containing the cleaved amyloid ß peptide were one of the dominant pathological hallmarks of postmortem AD brains, led to the amyloid cascade hypothesis, which proposed that amyloid was the direct cause of dementia, and therapies that would reduce amyloid accumulation would abrogate cognitive loss [126, 127]. Transgenic animal models carrying the mutated human APP and/or human mutant variants of the PS1/PS2 genes were generated and shown to accumulate amyloid plaques that looked similar to human Aß accumulation in AD brains. Use of active or passive anti-amyloid therapies in these animal models of AD showed reduced plaque pathology and prevented loss in behavioral task performance. However, these results did not translate into effective treatments for human AD [128, 129]. Disappointing results from anti-amyloid beta (Aβ) therapies or BACE inhibitor trials suggest that pursuing these therapeutic targets alone is likely to be unsuccessful. In addition, a number of cases of cognitively normal individuals were found to have high loads of amyloid deposits upon postmortem examination of the brain. Thus, while amyloid may be required for Alzheimer’s disease (by definition) it is clearly not sufficient [130]. This has also been supported by observations in multiple AD mouse models [86, 131, 132].

In contrast to the early-onset AD cases, most AD cases develop after the age of 65. Indeed, age is the greatest risk factor for the disease (although older age alone is not sufficient to cause AD dementia), with incidence doubling every 5 years and ultimately affecting ~ 32% of people 85 and older [125]. Genome-wide analysis of AD-associated polymorphisms for late-onset Alzheimer’s disease (LOAD, > 97% of AD patients) has identified genes involving immune processes and lipid metabolism, along with amyloid peptide and hyperphosphorylated tau accumulation, as key predictors of development of the disease [133]. ApoE genotype (ε4/ε4) has been identified as the greatest genetic risk factor for LOAD. Around 40–65% of Alzheimer’s patients have at least one copy of the ApoE4 allele (reviewed in [134]). Specific single-nucleotide polymorphisms (SNP) in the genes for clusterin (CLU), phosphatidylinositol-binding clathrin assembly protein (PICALM), and complement receptor 1 (CR1) were found to be associated with AD [135, 136]. Variants of TREM2, SPI1, and other genes that are significantly associated with AD have been subsequently confirmed as associated with the development and/or progression of disease [133, 137–139]. While the molecular mechanisms for the TREM2 variant disease association have yet to be determined, initial reports of an agonist anti-human TREM2 antibody has shown promise for slowing disease progression in mice [140]. The increasingly deep GWAS studies and refined differential gene expression data suggest that there may be many pathways that regulate the progression of Alzheimer’s disease pathology and the cognitive loss associated with the disease including the immune system and pathways involved in phagocytosis and clearance. Environmental factors, such as traumatic brain injury, diet, pollutants, or metabolic disorders, can then exacerbate deficiencies or overwhelm critical homeostatic checkpoints [141]. Consideration is now being given to the development of disease-modifying drugs such as those that can reduce excessive inflammation and/or regulate appropriate clearance pathways in the brain.

Inflammation was identified early on as a component of AD pathology [142] (and reviewed in [143–145]). Likely either or both peripheral and CNS localized inflammation can contribute to the progression of AD [146–148]. However, broadly targeted steroids, non-steroidal anti-inflammatories, and antioxidants that are known to reduce inflammation, have had limited success in AD clinical trials and can be associated with substantial undesired side effects [147, 149], highlighting the importance of very specific and temporally staged therapeutic targeting. In 2013, using an unbiased integrative network-based approach, Zhang, Gaiteri and colleagues showed that immune- and microglia-specific networks including complement genes involved in phagocytosis and inflammation, and TYROBP, a regulator of TREM2 function, are significantly upregulated in human sporadic/late-onset AD and correlated with disease traits [150]. In addition, they demonstrated that in mouse models overexpressing TYROBP similar pathways were induced, reinforcing that it is possible to assess human pathways in engineered mice [150]. Evidence that inflammation may indeed contribute to amyloid accumulation rather than just respond to it is increasingly compelling, including a recent study demonstrating that the Interferon γ induced IFITM3 protein actually enhances γ secretase activity resulting in increased Aβ production in cultured cells, and when knocked out amyloid plaques were reduced in the 5xFAD mouse model [151]. However, some of the upregulated proteins/pathways may be protective responses to perceived or real injury. Thus, it is critical to be aware of the presence of beneficial as well as detrimental effects in these immune pathways, including complement, and thus requires care in selection of therapeutic targets [152].

### Association of complement components with AD

Fibrillar Aβ was found to activate both the alternative and classical complement pathways in the early and mid-1990s [153–155] in vitro, as do neurofibrillary tangles [4]. Complement cascade proteins such as C1q, C3b, C4b, and properdin are colocalized with fibrillar Aβ plaques in both human AD and mouse models of the disease [43, 156–159] demonstrating the activation of this system in vivo. The activation-induced terminal complement complex, C5b-9 (MAC), has also been found in human AD brain in areas containing fibrillar plaques and tangles [160]. The presence of the MAC demonstrates that complete activation of the cascade does occur within the AD brain and thus is evidence for the generation of C5a and C3a. While complement activation products C5a and C3a may bind to their receptors and recruit phagocytic cells to the plaque (the source of the generated C3a and C5a), the intracellular signaling of C5a (and C3a) on microglia is predicted to synergize with plaque fAβ ligation of the TLRs inducing a chronic inflammatory state mediated substantially by locally produced pro-inflammatory cytokines as well as other neurotoxic species (reviewed in [161]). Ablation of C1q in the transgenic hAPP Tg2576 mouse model supported such a scenario as the absence of C1q, and thus inability to generate the downstream classical complement pathway products C3a and C5a, resulted in significant and substantial decreases in microgliosis and astrocytosis and prevention of synaptophysin and MAP2 loss in the CA3 region of the hippocampus [91].

As mentioned above, most complement factors can be synthesized within the brain and have been shown to be elevated during progression to AD (reviewed in [162, 163]), likely as a general response to injury that occurs in many neurological disorders. C1q was found to be dramatically increased in the normal aging of mouse and human brain [41]. Three pivotal reports in 2015, 2016, and 2017 revealed noncanonical activities of the classical complement components in aging and AD mouse models. First, Lemere and colleagues demonstrated a region- and age-dependent decrease in synaptic puncta density, spine density, neurons, LTP, and cognition in wild type mice that was not seen, or not so pronounced, in age-matched C3 knockout mice [164]. Hong et al. demonstrated the upregulation of C1q in the brain as early as 1 month of age in the J20 AD mouse model relative to wild type (similar to the upregulation of C1q mRNA at 2 months of age in 3xTg transgenic model [58]) prior to the accumulation of amyloid plaques [67]. Importantly, synapse loss could also be seen prior to amyloid plaque formation in both J20 and APP/PS1 mice at 3–4 months of age selectively in CA1, CA3, and dentate gyrus. An increase in C1q and C3 tagged synapses accompanied the decrease in synapse density [67]. This loss of synaptic puncta was not seen in APP/PS1 mice that were genetically C3 deficient. In addition, injection of Aβ oligomers also induced decrease of synaptic density and enhanced microglial engulfment of synaptic material within 72 h in wild type mice but not in C1q knockout mice, mice co-injected with the oligomeric Aβ and anti C1q, or mice deficient for iC3b/C3b receptor CR3 [67]. Finally, Shi and Lemere and colleagues reported that C3 constitutive knockout in the APP/PS1 mouse showed protection from synaptic loss as detected by high resolution confocal microscopy, and prevention of cognitive decline, reduction of proinflammatory cytokines and reduced gliosis even without a decrease in amyloid load [131]. These data clearly demonstrate that the early components of the complement cascade (C1 through C3) mediate region-specific synapse elimination both pre-plaque accumulation and at later ages when fibrillar amyloid plaques are prevalent.

While classical pathway activation clearly mediates synaptic pruning, this activation also generates C3a and, if C5 is present, C5a. These activation fragments, signaling through their respective receptors, can promote local neuroinflammation and neurotoxicity. The genetic deficiency of C5 has been shown to be one of a limited number of genetic differences associated with decreased amyloid deposition in DBA/2 J mice vs. C57Bl6 mice transgenic for the human APP gene [165]. Pharmacologic inhibition or genetic ablation of the C5a-C5aR1 interaction, while maintaining all upstream the complement components involved in synaptic pruning, rescued cognition and reduced AD pathology. Specifically, treatment with a C5aR1 antagonist, PMX205, significantly and substantially limits pathology in two mouse models of AD (Tg2576 and 3xTg) and trended toward rescue of a behavioral deficit [94]. In another proof of principle, immunization of Tg2576 mice with peptides mimicking the C5a C-terminus resulted in high titer antibody to C5a, and partial protection of contextual memory (~ 50%) [166]. In addition, CD45 reactivity and amyloid deposits were also reduced ~ 30–45% [166], similar to the effect seen in this model treated with the C5aR1 antagonist, PMX205 [94]. In another transgenic AD model, that contains a third “Arctic” mutation in the human APP transgene that accelerates the formation of fibrillar Aß plaques [167], genetic ablation of C5aR1 prevented the loss of neuronal complexity (CA1 region of the hippocampus) and the decline of cognitive performance seen in the C5aR1 sufficient Arctic mice at 10 months of age without any change of plaque accumulation [86]. These data are consistent with a detrimental role of C5a through its interaction with C5aR1. Since brain C5aR1 is predominately expressed on microglia and is upregulated in models of AD, the effect of C5aR1 signaling on microglial gene expression was investigated at 2, 5, 7, and 10 months of age [86]. While significant and substantial behavior differences were detected only at 10 months of age in this model, microglial transcriptome analysis identified inflammation-related genes as differentially expressed (DE) as early as 5 months of age, with increased expression in the Arctic mice relative to wild type. Importantly, this inflammatory expression profile was decreased in the Arctic mice lacking C5aR1 (Arctic/C5aR1KO) relative to Arctic. These upregulated genes include NFκB subunits which may contribute to increased NF-κB signaling and to the observed greater expression of inflammatory cytokines (Il-1α) and chemokines (Ccl3, Ccl4, Cxcl2), all of which were increased in the Arctic mice, but not so elevated in the Arctic/C5aR1KO [86]. In addition, phagosomal-lysosomal gene expression was increased in the Arctic mice but further increased in Arctic/C5aR1KO mice. CD68, TREM2, TYROBP, Fcgr4, cathepsins, glycosidases, and other enzymes that are involved in the endosomal-lysosomal pathway, showed significantly higher and earlier expression in Arctic/C5aR1KO microglia relative to Arctic. These include Aß-degrading enzymes that have been implicated in clearance of Aβ in vivo and those that degrade ingested apoptotic cells and neuronal blebs. Early “activation” of microglia cells is reported to be beneficial in AD models [141, 168]. However, as fAß plaques increase with age, microglial cells can become dysfunctional, and thus, the phagocytosis and clearance of Aß is reduced [169] while inflammation increases. C5aR1 deficiency appears to prevent polarization towards increased detrimental disease-associated inflammatory microglia seen in AD mouse models [86] while enabling continued phagocytosis and degradation with lower inflammatory sequelae. These microglial profiles are consistent with the different subtypes of microglia recently identified by single-cell RNA-Seq analysis in other AD models [40, 140, 141, 170] and suggests that inhibition of C5aR1 could result in preventing or slowing the induction of detrimental proinflammatory microglial subsets, perhaps by preventing the downregulation of “inhibitory-checkpoint pathways” [141], while enabling the nonphlogistic clearance of damaged synapses, apoptotic cells, or neuronal blebs.

As mentioned above, the absence of C3 by genetic ablation in AD models (or functional inhibition in other disorders [78]) has been linked to suppression of synaptic pruning, due to a lack of the classical pathway opsonization fragments, C3b and iC3b. However, during this process, the C3a fragment is also generated which is functionally active by binding C3aR. While C3aR gene expression is elevated in the brain of human AD and some tauopathies as well as in mouse models of AD and tauopathies [171], the role of C3a and C3aR has been elusive in Alzheimer’s disease. Since there has been some confusion in this literature, it is important to clarify that C3 does not bind C3aR, but rather the C3a fragment, which is generated when any of the complement pathways are activated and C3 is cleaved, is a functional C3aR ligand. In addition, several studies have used a “C3aR antagonist” that has serious caveats such as “agonist” properties at the high concentrations often used in vitro, as well short half-life in vivo [172], and thus conclusions from those studies must be supported using other reagents or design. C3aR expression is predominantly expressed in microglia and is strikingly upregulated in a tauopathy mouse model (PS19 tau) [171]. Complete genetic deficiency of C3aR in this model markedly abrogated the increases in microgliosis, astrocytosis, astrocyte C3, proinflammatory cytokines, hyperphosphorylated tau, behavior deficits, and LTP induction [171]. The lack of C3aR also rescued synaptic loss in CA3 and prevented decreased NeuN+ in CA1 and CA3, suggesting that the neuroinflammatory state resulting from stimulation of C3aR induces a neurotoxic environment. This report provides compelling evidence for a significant and substantial influence of the C3a-C3aR signaling in the progression of neurodegeneration in this tau model with conserved C3aR-controlled networks in human AD [171]. However, a recent study by El Gaamouch and colleagues reported that a peptide derived from cleavage of VGF, TLQP-21, signals via C3aR to enhance motility of and phagocytosis by microglia in vitro, reduces amyloid plaques and markers of dystrophic neurites, and reverses induction of AD genes in male 5xFAD mouse [88]. Similar responses were observed in vitro with a 15 amino acid peptide C3a superagonist, but not when the peptides were used on microglia or animals genetically lacking C3aR. Whether the sex-specific effects are due to the slower accumulation of pathology in male 5xFAD than females and/or the time of treatment administration or actual sex-specific differences remains to be seen. In addition, whether these two seemingly opposite outcomes are due to the difference between a model of tauopathy versus amyloidosis, consequences of developmental genetic ablation or other effects will also require further studies. Furthermore, how the balance of proposed C3aR and C5aR1 activities dictates functional outcomes over the course of the disease is also an area for additional investigation. However, the fact that in human AD reduced VGF has been identified as a driver of AD pathology and cognitive decline, and thus as neuroprotective [173], suggests tweaking the C3aR receptor may have therapeutic potential.

In addition to the amyloid-induced TLR signaling that results in NF-κB activation mentioned above, the NLRP3 inflammasome has been linked to the innate immune response to Aβ contributing to the elevation of IL-1ß production [174]. The NLRP3 inflammasome is a caspase-1 dependent signaling cascade that generates IL-1β. NLRP3 activation and IL-1ß levels are known to increase with age and correlate with increasing Aβ deposition in AD patients [175, 176]. Moreover, NLRP3 can be activated by both fAβ and low molecular weight Aβ oligomers and protofibrils suggesting that innate immune responses can be initiated before the onset of amyloid plaque deposition [177]. In the APP/PS1 mouse model of AD, knocking out NLRP3 resulted in increased phagocytosis of Aβ, reduction of Aβ pathology, a rescue of LTP deficits and spine alterations, and prevention of behavioral deficits [175]. Increased synthesis of NLRP3 components and proinflammatory products of the inflammasome is also a prominent feature of the human disease [178]. Thus, limiting the NLRP3 signaling cascade or the downstream cytokine response (IL-1β) has long been suggested as a potential therapeutic strategy for AD. C5a was found to increase the release of IL-1β and IL-6 when added to Aβ-primed human monocytes in vitro [179] and C5aR1 antagonists have been found to reduce proinflammatory cytokines (IL-1β) in periodontal disease [180]. Thus, it is possible that in the CNS, as in the periphery, C5a via C5aR1 signaling synergizes with TLR/NFκB and the NLRP3 inflammasome activity to trigger potent detrimental inflammatory responses [161]. If so, it would add further value to the use of a C5aR1 antagonist in AD, avoiding the systematic suppression of NLRP3, which could lead to susceptibility to infection.

**Fig. 2:**
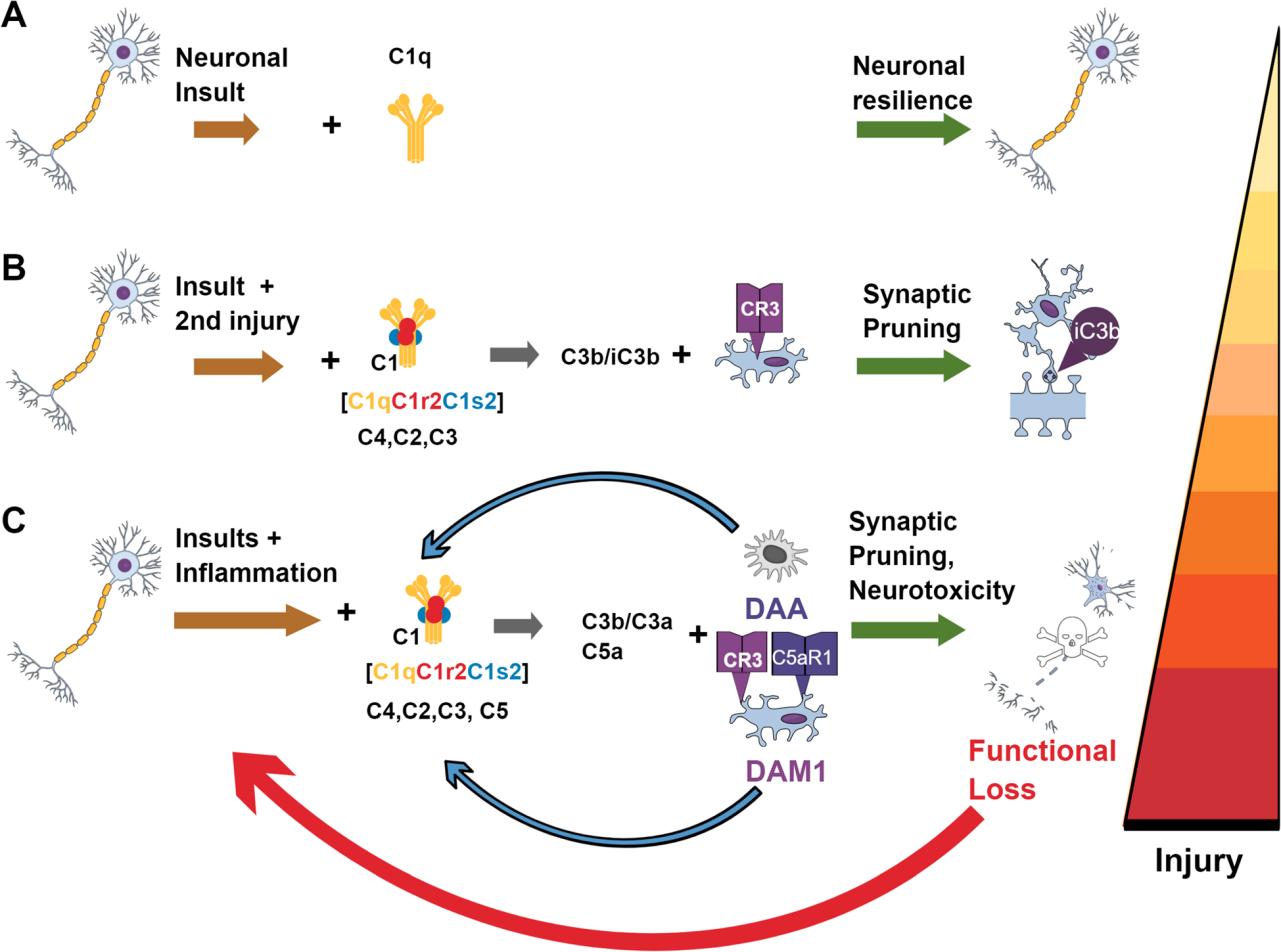
Complement contributions to neuronal health, synaptic turnover, and self-perpetuating feed-forward inflammation and neuronal dysfunction in the CNS. (A) C1q synthesis is rapidly induced upon perturbation. C1q engagement with neurons after neuronal insult in the absence of the proteases C1r and C1s, promotes neuronal resilience. (B) As additional injury occurs, additional complement components C1r, C1s, C4, C2, and C3 are synthesized resulting in the formation of C1 complex, C1q, C1r_2_, and C1s_2_, and cleavage of C3 to C3b/iC3b, which promotes synaptic pruning by microglia via the CR3 receptor. (C) Further insults promote inflammation and complement activation. Once C3 is cleaved, if C5 is present, C5a is produced along with C3b and C3a. Inflammatory damage-associated microglia (DAM1) and damage-associated astrocytes (DAA) [141] are induced and promote neurotoxicity resulting in functional loss of neurons and eventual neuronal death. Neuronal death and/or overwhelmed clearance mechanisms further promote inflammation to continue the cycle of injury. DAAs and DAMs interact with DAMPS/PAMPS and complement C5a and C3a to perpetuate neurotoxicity

Many gaps remain in our understanding of the consequences of synapse loss and consequent decrease in LTP in the pre-plaque stage of the disease, of synapse loss in the plaque-associated stages, and of which (or all) complement activation events actually contribute to cognitive dysfunction. Further investigation of the correlation of synaptic pruning activity, which, as mentioned above, is also involved in plasticity [63, 181], is necessary. It is likely that neuroinflammation plus excessive synaptic pruning participate in an ever-increasing cycle of glial activation, neurotoxicity/damage, complement activation, and synapse elimination that together overwhelm the neuronal resilience to perturbation, and thereby lead to loss of cognitive function (Fig. 2).

### Regulators of complement

CR1 is a cell-associated regulatory protein, which binds C3b (and more weakly to C4b and C1q). In humans, CR1 on red blood cells plays a major role in the clearance of C3b-opsonized immune complexes via the mechanism called “immune adherence.” Activation of the classical cascade results in C3b bound to immune complexes (IC-C3b) via its thioester bond. IC-C3b binds to erythrocyte CR1 which then transports the immune complexes to the liver and spleen for degradation and clearance [182]. Activation of either the classical or alternative pathway by fAβ results in the covalent association of C3b with the amyloid fibers, as discussed above. In GWAS studies, polymorphisms in CR1 have been repeatedly associated with AD risk. Keenan and colleagues identified a SNP that is within the coding region of CR1 [183] resulting in an amino acid change (S1610T) within the protein domain that has been attributed to C1q binding [184]. However, that SNP cosegregates with another polymorphism that decreases CR1 density on red blood cells [185, 186] and thus may result in less efficient clearance of complement-opsonized Aß from blood. Thus, the possibility that red blood cell CR1 could be involved in clearing peripheral Aß was initially tested in a study of 36 individuals. AD patients had significantly lower levels of C3b-opsonized Aβ bound to their erythrocytes than age-matched controls or mild cognitively impaired individuals consistent with a defect in peripheral amyloid clearance mechanisms [187]. Additional studies by Rogers and colleagues demonstrated that red blood cells from AD patients had less associated Aß compared with cognitively normal individuals and that Aß and red cells were colocalized with liver Kupffer cells [188–190] as would be expected if CR1 played a role in Aß clearance from blood and thus contributed to a peripheral sink for Aß [191]. Taylor and colleagues propose an interesting testable therapeutic strategy consisting of anti CR1 and anti Aß bispecific antibodies that would promote Aß clearance from the blood and thus enhance the peripheral sink to reduce Aß load in the brain [192]. While CR1 and CR2 are coded for by distinct genes in humans, mice express CR1 and CR2 proteins that result from differential splicing of a single Cr2 gene. Crry, a closely related gene, functions similarly to CR1 in the regulation of C3b amplification, but is structurally very different (reviewed in [193]). In addition, CR1/Crry in the mouse is not expressed on erythrocytes as in humans, making mouse models of the role of red blood cell CR1 in AD more challenging. However, a transgenic mouse expressing CR1 in mouse erythrocytes [194] exists, and a new model with a human CR1/CR2 knock in created by the MODEL-AD consortium [195] may eventually enable a better understanding of the role of this regulatory protein in protection from Alzheimer’s disease.

Factor H and C4-binding protein (C4BP) are soluble complement regulators that limit the alternative and/or classical pathway C3 and C5 convertases respectively. C4BP is a cofactor for cleavage of C4b by factor I, leading to inactivation of C4b and thus inhibiting classical and alternative pathway C3 and C5 convertase activity, an activity also shared at cell membranes with factor H. C4BP is colocalized with plaques and dead cells (TUNEL staining) in AD brains and binds Aβ in vitro, *c*onsistent with a role in reducing a proinflammatory response induced by ongoing complement activation and generation of C5a [196]. Another regulator of proinflammatory effects of complement activation is carboxypeptidase N (CPN) which rapidly cleaves the C-terminal arginine from C3a and C5a thereby reducing their affinity for their cell surface receptors and thus reduces pro-inflammatory actions. As of yet, little is known of the expression and role of CPN in the CNS [197].

All of the above proteins contain consecutive multiples of a common motif known as complement control proteins (CCP), or short consensus repeats or Sushi domains. Use of protein sequence and structure databases have uncovered multiple other proteins with these characteristic motifs (reviewed in [8]). Many of these are expressed in the brain, some with genetic association with neurological disorders [198, 199] and are currently being investigated as potential endogenous complement regulators. One such “sushi repeat protein,” SRPX2, has been characterized as increasing synapse density [200]. Mutations in this X-linked SRPX2 are associated with language disorders. Sia and colleagues recently extended these studies to demonstrated that this protein inhibits complement-dependent synapse pruning during postnatal development with region- and synapse-type specificity [7]. Understanding the molecular basis for the induction of expression and the inhibitory specificity of these newly uncovered families of proteins in the brain may provide pathways to precision therapeutics to control synaptic vulnerability to elimination.

### Potential of complement components as biomarkers in AD

Many have explored the possibility that complement components may provide a biomarker much desired in the field (Table 3). An impressive meta-analysis of 86 such studies showed a significant increase in individuals with AD of only CLU and C3 in the spinal cord fluid (CSF) with inconsistent elevations in plasma C1q and factor H [216]. However, the Cardiff group found that a model considering plasma levels of 3 components (CLU, factor I, and C5b-9) was predictive of conversion from mild cognitive impairment (MCI) to AD over a 1-year period [219], and more recently discovered that after adjusting for age and APOE4, other combinations of complement components had some value (AUC of 0.71) in predicting progress to AD [215]. The difficulty in achieving a pan-AD higher degree of correlation with specific protein biomarkers may be inherent in the various pathways that contribute to the development of AD as indicated by the diverse genetic risk factors and the environments that synergize with the polygenic risk paths in diverse ways. Recently, there have been promising reports correlating complement activation cleavage components in plasma astrocyte-derived exosomes (ADE) with AD disease progression [201], but additional confirmatory work is needed.

**Table 3:** Complement as a biomarker for neurodegenerative diseases

	AD	ALS	Stroke	Epilepsy	TBI	MS
C1	C1q elevated in plasma enriched for astrocyte-derived exosomes (ADE) early in AD [201]	Elevated in serum [82]				C1q elevated in plasma ADE [202]
C4	C4a is elevated in plasma [203], C4b elevated in ADE [201]	Elevated in serum [82]	C4d elevated in plasma 24 hr after stroke [204]	C4 is elevated in patients with uncontrolled seizures vs controlled [205]	C4b chronically elevated in plasma ADE in mild TBI [206]	C4/C4a increased in plasma and CSF [207, 208]
C3	C3d elevated in ADE [201]	C3 Elevated in serum [82]. C3a unchanged in plasma of ALS patients compared with age-matched controls [209]	C3a elevated in plasma 1-14 days after stroke and correlated with prognosis [82, 204, 210]	Properdin & C3 elevated, iC3b decreased in serum of patients with uncontrolled seizures vs controlled seizures [205, 211]	C3b chronically elevated in plasma ADE in mild TBI [206], C3a elevated in serum immediately after injury and remains chronically elevated [212]	C3b/iC3b elevated in plasma ADE [202], and in whole plasma [207]. Properdin elevated in CSF [208]
C5		C5a elevated in the plasma of ALS patients [209]	C5a elevated in plasma 1-14 days after stroke [82, 204]		Serum C5a increases immediately after injury & continues to rise up to 10 days post injury [212]	C5/C5a elevated in plasma ADE [202]
C5b-9	C9 is elevated in plasma [203]. C5b-9 elevated in ADE [201]. sC5b-9 decreased in patients with MCI who later develop AD [213]	Elevated in plasma of ALS patients [209]	Plasma sC5b-9 is elevated after stroke and increases throughout recovery, but is decreased in mild cases [82, 210, 214]	C5b-9 elevated in plasma of patients with focal and generalized seizures, clusterin reduced [205]	Elevated in plasma ADE in mild TBI cases for up to 12 years after injury [206, 212]	C9 levels reduced in plasma and CSF of MS patients [207]
Inhibitory factors	FI decreased in patients with MCI who later develop AD [213, 215]. Clusterin is elevated [217], CD59, CD46, CR1, DAF decreased in ADE [201]			fH elevated in plasma of patients with focal and generalized seizures, clusterin decreased in patients with uncontrolled seizures vs controlled [205]	FI, CR1, CD59, C4BP decreased shortly after injury in plasma ADE [206, 212]	fH elevated in plasma ADE [202], C1inh and fH elevated in plasma and CSF [207, 208]
Factor B	Elevated in ADE [201]					fB reduced in CSF and plasma [207]

## Amyotrophic lateral sclerosis

Amyotrophic lateral sclerosis (ALS) is a neurodegenerative disease that is characterized by the progressive death of upper and lower alpha motor neurons in the motor cortex, brain stem, and spinal cord resulting in muscle weakness, paralysis, and death. Diagnosis typically occurs between ages 54–69, and risk factors include age, male sex, and familial occurrence of ALS [220]. The majority of ALS cases are sporadic (90%) with no known etiology, while the remaining 10% are familial [82]. Annual incidence of ALS is estimated to be between 0.6 and 3.8 per 100,000 persons, with death occurring 24–50 months after onset of symptoms [220]. Mutations in the copper-zinc superoxide dismutase 1 (SOD1) gene are associated with familial ALS. The SOD1^G93A^ mutation was used to develop the predominant mouse model of ALS. This mutation causes misfolding of the SOD1 protein, resulting in protein aggregates containing TDP-43 that can activate microglia and inflammatory pathways [221]. Evidence suggests that ALS disease pathology begins in the peripheral neuromuscular junction, and when neurons lose their connection to muscles, the motor neurons atrophy thus spreading degeneration to the CNS in a “dying-back mechanism” [222].

Several studies have provided evidence of complement activation in ALS that may contribute to both initiation and progression of the disease. In ALS patients, circulating levels of C1q, C3, C4, C5a, and C5b-9 were found to be elevated, indicating both increased capacity and recruitment of the classical and terminal complement pathways [82, 209]. As seen in postmortem sections of spinal cords of ALS patients, C1q and C3d proteins colocalized with GFAP-positive astrocytes and HLA-DR-positive microglia, while proteins associated with MAC were localized only to microglia. Furthermore, C1q and C4 mRNA was upregulated in neurons and glia in the spinal cord compared with controls, suggesting a local source of complement components in the CNS [32].

Complement gene upregulation is also observed in SOD1 and TDP43 transgenic mice [33, 82]. RT-PCR of isolated motor neurons from SOD1 mice at pre-symptomatic disease onset revealed dysregulation of 108 genes, which can be divided into functional groups including neuronal regeneration/injury, lysosomal degradation, apoptosis, and complement pathway [33]. Within the complement system, C1q(a-c), C5aR1 mRNA is upregulated in mouse models of ALS [33, 223]. Microdissection and in situ hybridization confirmed that C1q and C5aR1 mRNA is upregulated in spinal motor neurons. In the SOD1 and TDP-43^Q331K^ models of ALS, quantitative RT-PCR analysis revealed increases in C1qB and C4 mRNA, suggesting recruitment of the classical pathway [224, 225]. Immunohistochemical experiments confirmed that C1q and C5aR1 were localized to motor neurons and microglia [224]. Furthermore, regulatory complement components CD55 and CD59a were upregulated throughout disease progression in the lumbar spinal cord and tibial skeletal muscle, suggesting an attempt at regulation [96, 224]. In a rat model of ALS, C3/C3b immunoreactivity was increased in the ventral lumbar spinal cord and localized to motor neurons and astrocytes. Similarly, C5aR1 immunoreactivity is primarily localized to motor neurons in WT rats but increased in astrocytes in end-stage ALS rats [97]. Interestingly, C3 mRNA is only increased in the later stages of disease, and C3 protein localized to motor neurons and microglia. These findings suggest cellular- and temporal-specific roles of complement components in ALS pathology.

Sequential deletion of complement components has delineated the roles of different components in ALS disease progression. Deletion of upstream complement proteins C1q, C3, or C4 did not alter onset or severity of symptoms, including micro- and astro-glial gene expression or motor neuron loss [82, 95]. However, genetic deletion or pharmacologic blocking of the downstream C5aR1 results in improved outcomes, including reduction of macrophage infiltration and denervation, and increased grip strength and survival, suggesting that a newly defined extrinsic complement pathway may play a role [96–98]. C5aR1 levels are increased in the spinal cord and localized to motor neurons and glia. Blocking C5aR1 genetically or pharmacologically in SOD1 mice delayed disease progression and improved survival and motor function [82, 96]. Treatment with the C5aR1 antagonist PMX205 slowed disease progression in a model of ALS in both mice and rats, delaying decline of grip strength and prolonging survival compared with vehicle-treated animals [97, 99].

## Stroke

An ischemic stroke occurs when an artery that provides blood to the brain is blocked. Ischemic stroke is often followed by reperfusion injury once blood flow is restored. During ischemia, there is a buildup of xanthine dehydrogenase, which interacts with oxygen when blood flow returns that subsequently leads to production of superoxide, hydrogen peroxide, and hydroxyl radical OH, all leading to endothelial activation and release of proinflammatory cytokines [226]. This can lead to a secondary injury caused by inflammation, oxidative stress, and/or BBB breakdown [227], which can in turn cause exacerbation of tissue damage, neurological deficits, and cognitive impairment. Thus, stroke injury can present itself with an acute phase (within 24 h), subacute phase (1–5 days), and chronic phase (weeks to months). Clinical studies showed that complement components C4d, C3a, C5a, and C5b-9 are elevated in the serum of patients during the subacute phase (24–48 h after stroke) [204, 214]. A follow-up showed that the terminal complement pathway remains upregulated in the serum of patients 7 days after stroke [214], consistent with chronic complement activation. C5b-9 protein levels were significantly correlated with volume of ischemic infarction and measures of neurological deficits in stroke patients [204, 214]

Stroke is induced experimentally in animal models often by occlusion of the middle cerebral artery (MCAO), and stroke-like conditions can be induced in vitro with glucose/oxygen deprivation in cell culture. Experimental models of stroke result in elevated levels of complement components (C1q, C3a, C5a) in the brain [23, 36, 106, 228, 229] and upregulation of C1q mRNA in microglia [23] and C5 mRNA in neurons [36]. Immunohistological evidence support both a protective and deleterious effect of complement after stroke [228, 229]. Although stroke results in substantial BBB damage, RT-PCR evidence from primary neuronal cultures suggests that complement components such as C5 and then C5a can be produced by neurons and are upregulated after ischemic injury [36]. Complement activation acts synergistically with other inflammatory mediators (e.g. TLR-4) to create an amplified immune response. V-set immunoglobulin in domain containing 4 (Vsig4), also called CRIg, is a membrane protein expressed on macrophages that regulates complement-mediated inflammation by binding C3b to promote clearance of opsonized fragments [230]. CRIg, also expressed by microglia, is downregulated after stroke, reaching its lowest expression at 3 days post injury. Overexpression of CRIg prior to MCAO or oxygen-glucose deprivation is neuroprotective, reducing ischemic volume, preventing TLR-4-induced inflammation, and attenuating neuronal loss [231].

After ischemia, C1q is produced locally by microglia [23] and is associated with neurons as early as 6 h after injury [232]. C1q deficiency or treatment with C1-inhibitor significantly reduces infarct volume, inflammatory cytokine production, and BBB permeability following transient MCAO [100, 102]. There is some evidence the C1 Inhibitor may act independently of the classical complement pathway to prevent injury via suppression of the contact-kinin system, thereby reducing expression of adhesion molecules in a complement-independent manner [102, 233]. Nevertheless, hypoxic-ischemic injury in C1q knockout mice have reduced macrophage activation and C3 deposition in the ischemic brain compared with WT, suggesting that downstream complement activation is involved in stroke pathology [100]. However, C1q promotes angiogenesis and tube formation after MCAO via interaction with the inhibitory collagen receptor LAIR-1 [101]. In addition, MBL knockout mice have reduced ischemic volume after MCAO and less deposition of C3 in the brain parenchyma, suggesting involvement of the lectin pathway [103]. Neurological outcomes were also improved in these mice relative to wild type. Mice deficient in both C1q and MBL (C1q/MBL KO), or only deficient in factor B or factor H (alternative pathway) had better neurological outcomes and preservation of neuronal integrity [105], suggesting that the alternative pathway may amplify the effects of the classical and lectin pathways after MCAO. Furthermore, there was no detectable C3 deposition in C1q/MBL KO mice, suggesting that activation of the alternative pathway alone is not sufficient to drive complement-mediated injury.

Evidence suggests that complement C3 is central to stroke pathology in animal models [234]. MCAO resulted in a significant increase in mRNA of C3a and C5a receptors on the ipsilateral side [35], and reports have shown that C3aR and C5aR1 mRNA peaked at 24–48 h after injury [35, 235]. C3aR is overexpressed in the subventricular zone and localized to infiltrating CD3+ T cells after MCAO [229]. Pre-treatment with a low-dose C3aR antagonist (C3aRA) reduces subcortical infarct volume, reduces mortality, and improves spatial memory and sensory-motor function [229, 236]. Treatment shortly after intracerebral hemorrhage accelerated neurological and cognitive recovery in mice [236]. In cell culture, oxygen-deprived endothelial cells treated with C3aRA have reduced cell death and activation of adhesion molecules (ICAM1), reduced phosphorylated ERK, and increased occludin, all suggesting preservation of BBB integrity [237]. It is important to note that the aforementioned C3aRA (SB290157) has been reported to have off-target activity, and in high doses may act as an agonist instead of antagonist [172]. However, C3 knockout mice also have reduced infarct volume after MCAO and improved neurological function [106], making C3-mediated activities a good therapeutic target. In contrast, there is some evidence that C3a may promote neurogenesis and plasticity after stroke. Immediately after MCAO, there is a transient decrease in C3a receptor mRNA [35]. Overexpression of C3a under the GFAP promoter upregulates GAP43 production, an axonal marker indicative of neurite extension, after stroke [228]. C3aR knockout mice have a reduction of this marker compared with WT and C3a overexpressed mice, suggesting that C3a promotes neurogenesis and synaptic plasticity following stroke [228]. In vitro, C3a treatment enhances survival of astrocytes and attenuates expression of glial fibrillary acidic protein after ischemia [238]. Furthermore, intranasal treatment with C3a one week after stroke significantly improved motor function in the grid walking and cylinder tests [228]. Clearly, additional experiments using C3aR−/− (preferably conditional and inducible) and/or more specific C3aR antagonists are essential to translate these findings to the clinic. This is particularly important, as recently, complement inhibition targeted to ischemic lesion with an injury-targeted C3b inhibitor, B4Crry, while significantly reducing C3a levels in the brain also reduced infarct volume and neurological score up to 30 days after injury [107]. B4Crry treatment, acting to dissociate the C3 convertase and prevent further C3b deposition, improved performance in hippocampal-dependent memory tasks (Barnes Maze) and attenuated C3b-mediated microglial engulfment of stressed or active neurons. Gene expression analysis revealed that this C3b-targeted complement inhibition downregulated inflammatory genes and upregulated complement inhibitor genes. Since suppression of C3 convertase activity also limits the production of C3a, both C3a and C3b may mediate acute and chronic effects of MCAO [107]. These data suggest a dual role of complement activation after stroke, wherein C3 activation products can promote recovery and/or contribute to neuropathology. Thus, manipulation of C3-mediated activities after stroke would have to be highly targeted and temporally regulated.

Acute effects of MCAO include upregulation C5a receptor mRNA [35]. At 6 h post-MCAO in a mouse model, C5 mRNA increased 6-fold compared with sham-operated controls, and by 24 h, it increased 8-fold (and 23-fold compared with naive controls) [35]. Increase in complement C5a after oxygen-glucose deprivation promotes cell death via apoptosis in cultured neurons. Experiments in which either cultured astrocytes or microglia were treated with oxygen-glucose deprivation resulted in no or very slight increase in C5a production, supporting that neurons are the primary source of C5 in ischemic conditions [36]. Cultured neurons that have pharmacologic or genetic block of C5aR1 show increased survival and fewer apoptotic cells. Mice lacking C5aR1 have displayed smaller infarct sizes after stroke and have better neurological outcomes than their WT controls [36]. However, other studies that blocked MAC formation with C6 KO or CD59a KO mice did not result in neuroprotection after MCAO [105]. This supports that inflammation triggered by C5a-C5aR1 interactions may promote ischemic injury, but C5b-9-induced cell lysis does not substantially contribute to stroke pathology.

## Epilepsy

Epilepsy is a neurological disorder characterized by spontaneous seizures that, broadly defined, is caused by an imbalance of excitatory and inhibitory synapses. Some patients with epilepsy have genetic mutations that cause developmental seizures during early infancy/childhood (for instance sodium channel mutations) [239]. However, some epilepsies are acquired after events such as traumatic brain injury, infection, fever, or drug use/withdrawal. Epilepsy affects approximately 65 million people worldwide, and 20–30% have uncontrollable seizures. The evidence of inflammatory mechanisms as a cause and consequence of seizures in epilepsy has been vastly reported [240]. Early studies showed increased levels of C3 in the serum in individuals with epilepsy compared with healthy controls and to patients with epilepsy that were receiving treatment [211]. This and other early studies have stimulated some interest in investigating the role of complement in human and animal models of epilepsy. The use of surgically removed brain tissue from individuals with drug-resistant seizures has allowed for histological and biochemical analyses. In samples resected from people with temporal lobe epilepsy (TLE), increased C1q and C3 gene expression has been reported [241]. Furthermore, C1q and C3c/C3d proteins were found localized to microglia, astrocytes, and neuronal somas [241]. Microglia also localize to regions with decreased Map2 immunoreactivity, suggesting a role for microglia in dendritic instability in epilepsy, perhaps by promoting aberrant phagocytosis of injured yet viable neurons through complement-mediated mechanisms [242]. To further investigate this possibility, Brewster and colleagues determined the localization of complement proteins in relation to microglia and dendrites in resected brain samples from people with focal cortical dysplasia. They reported increased complement proteins C1q and iC3b, as well as altered levels of phagocytic proteins Trem2, MerTK, and protein S1. Furthermore, they observed C1q clustered to points of contact between microglia and dendrites [243]. These observational studies suggest a role for complement in seizure-induced neuronal injury. Thus, preclinical studies have focused on describing and inhibiting complement activation in models of acquired epilepsy.

Brain insults such as TBI, fever, or alcohol withdrawal can result in a prolonged, continuous seizure (status epilepticus; SE). SE greatly increases the risk of developing TLE after a seizure-free latent period, a process called epileptogenesis. In a rat model of TLE, gene expression of C1q, C1r, C4, C3, C3aR, C1 inhibitor, and CD59 were upregulated during epileptogenesis compared with controls. Most of these genes remained increased months after the initial insult, during the chronic epilepsy phase, suggesting that elevated complement protein levels may contribute to the “priming” or maintenance of an epileptic brain [241]. In a similar model, higher protein levels of C1q and iC3b were detected in the hippocampi of epileptic rats, which were also associated with increased Iba1 and decreased Map2, and hippocampal-dependent memory deficits [244, 245]. Increased C3 levels are also significantly correlated with frequency of spontaneous seizures [244, 246].

Several studies suggest mechanisms by which complement proteins play a role in the development of epilepsy. C1q is involved in the removal of redundant synapses during development [39, 64]. Genetic ablation of C1q results in increased cortical neuronal complexity and spine density with a bias towards excitatory synapses [108], hyperexcitability, and absence seizures [62]. Acute treatment with intravenous immunoglobulins (IVIg), which scavenge complement activation fragments C3b, C4b, C3a, and C5a, thus blocking their activity [247] after SE, reduces the number of spontaneous seizures in the chronic phase and levels of hippocampal C3 in the latent phase [109]. In a model of malaria-induced seizures, C5-deficient mice had significantly lower frequency of spontaneous seizures [110]. Furthermore, sequential administration of components of MAC (C5b-C9) results in spontaneous seizures [248]. These findings suggest that downstream activation of complement (MAC) is required or at least involved in epileptogenesis. However, it is possible that C5a, independent of MAC formation, increases susceptibility to seizures by promoting inflammation and microgliosis. Despite the emerging evidence that complement proteins play a role in the generation of seizures, few studies have targeted complement as a potential treatment to stop disease progression. Current antiepileptic drugs fail to change disease trajectory, thus highlighting the need for novel therapeutics. One study showed that chronically epileptic mice from the kainate model spent significantly less time having seizures immediately after treatment with C5aR1 antagonist PMX53 compared with vehicle-treated epileptic mice [111]. Treatment with PMX53 also reduces the likelihood of a second seizure in a kindling model [111], suggesting that blocking C5aR1 can have antiepileptic effects.

## Traumatic brain injury

Traumatic brain injury (TBI) is a complex disease that varies in cause, severity, and, consequently, prognosis [249]. TBI often results in a secondary inflammatory injury that can contribute to development of post-concussive syndrome or chronic traumatic encephalopathy [250]. Therefore, the window between the initial TBI and secondary injury is critical for prognosis and preventative treatment [251]. The Glasgow coma scale (GCS) has been the primary measure of severity; however, this scale is imperfect at determining prognosis, and occluding factors (such as sedation or alcohol consumption) can interfere with test results [252]. Therefore, much effort has been placed on identifying biomarkers specific for different severities of TBI.

The change in complement function after TBI is biphasic; complement hemolytic serum activity is significantly decreased immediately after TBI, and it gradually increases to baseline levels at 5 days post injury, but continues to increase relative to controls at 10 days post injury [212]. Whereas C3a levels immediately peak in the serum after TBI and remain elevated, C5a levels are increased immediately after injury but continue to rise substantially up to 10 days after initial injury [212]. A recent panel of serum-derived proteins from patients with mild, moderate, and severe TBI revealed proteins that are elevated in all levels of TBI compared with control subjects (serum amyloid A, C-reactive protein, LPS-binding protein), proteins that are downregulated in moderate and severe TBI compared with mild TBI and controls (kininogen), and proteins that are upregulated only in severe TBI cases (apolipoprotein E and zinc alpha-2 glycoprotein) [252]. Analysis of CSF may provide more accurate biomarkers for trauma in the brain. Exosomes isolated from CSF of patients with TBI had elevated levels of NLRP1 inflammasome proteins, cytokines such as IL-1β, and astrocytic markers GFAP and S100B relative to controls [253]. In plasma, astrocyte-derived exosomes (ADE) from patients with mild TBI had transient increases in C4b, C3b, and C5b-9 levels. Patients with moderate chronic TBI had sustained high levels of C4b, C3b, and C5b-9, which remained elevated over 12 years after the last injury [206]. Importantly, these studies reveal a decrease in complement regulatory proteins such as CR1, CD59, C4BP, and factor I shortly after injury [206, 212], suggesting that the increase in complement activation is not properly controlled after TBI. Evidence from surgically resected brain tissue after TBI suggests local synthesis of complement component C3 [254]. This, along with pre-clinical data discussed below, support that complement activation plays a role in secondary injury after TBI.

In preclinical models of TBI such as controlled cortical impact (CCI) injury or cortical contusion, there is evidence of BBB disruption, inflammation, microgliosis, astrogliosis, and neuronal loss that are all associated with poor long-term cognitive outcomes [255, 256]. Following TBI in rodents, there is a significant increase in microglia cells and synapse loss [113, 255]. Also observed are increased mRNA and protein levels of C1q, C3 and CD11b, and MAC (C5b-9) proteins [113, 255]. Interestingly, microglial engulfment of synaptic elements only increases at late, but not early, time-points after injury, suggesting that this is a mechanism of secondary injury that can result in cognitive decline and is a potential therapeutic target. Complement-mediated microglial engulfment of neuronal and synaptic elements has been proposed as a mechanism of pathology in several neurodegenerative diseases as discussed above.

Early studies supported the hypothesis that the terminal complement pathway contributes to TBI-induced neurological deficits and neuronal injury [257, 258]. The terminal pathway makes an attractive therapeutic target because its inhibition prevents the amplification of C3 and C5 convertase generation leading to the formation of MAC and thus tissue damage, while maintaining the majority of the complement components functional for potentially protective processes (such as C3a/C3aR-mediated neurogenesis) [259]. Components of MAC are elevated early after TBI and are associated with TBI-induced microgliosis, astrogliosis, and apoptosis [118, 255]. Inhibition of MAC formation after TBI reduces neurological deficits and weight loss and reduces gliosis, apoptosis, and neuronal damage up to 72 h after injury [117, 118]. However, chronic neurodegenerative and cognitive effects of TBI may not be evident at early time-points, therefore these effects may not offer long-term protection. In the CCI model of TBI, complement inhibition with the fusion proteins CR2Crry or CR2fH, both inhibiting at the C3b amplification step, restores dendritic arborization [116]. Complement inhibition after TBI also restores motor function [116] and cognitive performance [113, 116] even when delayed treatment of up to 1 month after injury. Ablation of the alternative pathway alone (factor B KO) is sufficient to reduce C5a serum levels to baseline [115]. MAC inhibition with a C6 antisense oligonucleotide (ASO) or CR2-CD59 treatment, on the other hand, only alleviates short-term effects such as motor deficits, weight loss, apoptosis, and microgliosis [116, 118], but does not prevent chronic impairments including memory deficits and glial scarring [116]. Inhibition of the alternative complement pathway alone is sufficient to reduce glial scarring, lesion size, and motor/cognitive deficits [116], suggesting that this pathway is primarily involved in TBI-induced injury.

## Multiple sclerosis

The etiology of multiple sclerosis (MS) is not completely understood, but it is generally accepted as an autoimmune disorder in which myelin is aberrantly recognized as a pathogen, resulting in inflammation, demyelination, and axonal damage [260, 261]. MS and other demyelinating disorders are characterized by loss of central and peripheral system myelination due to an autoimmune response and complement-mediated oligodendrocyte and Schwann cell destruction, leading to axon degeneration [262]. This inflammatory response is thought to be initiated by T cell infiltration into the CNS [261].

In addition to demyelination and axonal damage, there is emerging evidence that synapse and spine loss is a part of MS pathology [78, 263, 264]. There is new evidence suggesting that microglia engulf synaptic elements in the visual thalamus in MS patients [78]. Microglia can engulf synapses via complement receptor CR3 in development and disease [39]; thus, complement may be a mechanism contributing to synapse loss and neurodegeneration in MS. Astrocyte-enriched extracellular vesicles derived from the plasma of MS patients contained high levels of C1q, C3, C3b, iC3b, C5, and C5a compared with controls and were inversely correlated with synaptic proteins detected in neuronal-enriched extracellular vesicles [202]. Interestingly, this increase in complement cascade proteins was not detected in whole plasma, suggesting a specific role of astrocytes in MS-associated complement activation. Whole exome sequencing identified variants of 12 genes that co-segregated in MS in 34 families with multiple incidence of MS. Among the genes identified were MASP1 and complement C2. Aside from complement factors, other variants identified were associated with immunological pathways, supporting an autoimmune cause of MS [265]. In a study by Barnum and colleagues, levels of complement proteins C1q, C3b, C4d, C3aR, and C5aR, and FBb were upregulated in MS brains and spinal cords compared with controls, suggesting involvement of classical and alternative complement pathways [266]. Specifically, C1q and C3b immunostaining was localized to reactive GFAP-positive astrocytes, which may be indicative of complement-mediated ingestion. In MS tissue, microglia express higher levels of inflammatory mediators C3aR and C5aR, as well as with C1q in the areas surrounding lesions, consistent with the promotion of complement to an inflammatory state that contributes to neurodegeneration in MS [260, 266, 267]. Furthermore, reactive microglia localize to C3b-positive neurons and myelin in lesion sites, suggesting that complement may mediate neuro-immune interactions in MS lesions [267].

Similar to human studies, complement proteins are elevated in preclinical animal models of MS and localize to microglia, synaptic elements, and lesion areas [78]. In experimental autoimmune encephalomyelitis (EAE), a mouse model of MS, inhibition of the central complement protein, C3, with cobra venom factor (CVF) reduces severity of pathology [121]. Treatment with a soluble form of complement receptor 1 (sCR1) in a model of EAE attenuates inflammation and demyelination, although disease onset is not completely eliminated [121, 122]. These studies support a role for complement in EAE pathology and present the complement pathway as a potential therapeutic target. However, chronic administration of a potent complement inhibitor is not ideal. Therefore, recent studies have focused on targeting more specific and downstream complement components, and the effects of transient, as opposed to chronic, treatment.

Through systematic elimination of individual complement components, more precise therapeutic targets can be identified. Genetic deletion of C4, which is necessary for the formation of classical pathway C3 convertase, did not alter disease progression and even resulted in increased cytokine expression [119, 121]. Microglia-mediated synaptic loss has been observed in EAE models independently of demyelination in the hippocampus [268]. Increased C1q and C3 mRNA and protein have been observed in the hippocampi of EAE mice [269]. C3, but not C1q, colocalizes with engulfed synaptic elements, suggesting that the classical complement pathway is not required for in MS pathology [78]. Interestingly, C3 knockout, but not C1q knockout, significantly reduced EAE clinical score and preserved hippocampal synaptic density [269]. However, C1q deposition on cells observed in the brains and spinal cords of MS patients [266, 267] may indicate a non-canonical role of C1q in MS (or a small role for the classical pathway in disease progression). Genetic deletion of factor B (necessary to form C3 convertase in the alternative pathway) showed promising results with delayed onset and reduced severity of EAE symptoms [120]. Further support for the alternative pathway came from the use of an anti-factor B antibody (mAb 1379) [262]. Short-term treatment with mAB 1379 5–11 days after induction of EAE with pertussis toxin resulted in long-term reduction of clinical severity score, demyelination, and inflammation in the brain and spinal cord. These effects remained long after treatment ended and factor B levels would have been expected to return to baseline, suggesting that mAb 1379 treatment may permanently reduce inflammatory responses that promote demyelination in models of MS [262]. Additionally, short-term treatment directly following the onset of symptoms (days 9–15 post induction) in a transfer EAE model of MS significantly reduced EAE severity, suggesting that intervention after clinical signs arise may be beneficial. Further evidence supports that inhibition of the terminal complement pathway is neuroprotective in a model of chronic EAE [270]. Inhibition of MAC with a C6 ASO prevented relapse of clinical symptoms and demyelination in mice with chronic EAE and inhibited NLRP3 inflammasome activation, whereas inhibition of C5aR1 with PMX205 reduced inflammatory gene expression and only partially decreased NLRP3 inflammasome [270]. These data support that activation of the terminal complement pathway, possibly via alternative pathway activation, promotes inflammation and degeneration in experimental MS models.

## C5a-C5aR1 as a therapeutic target

There are several potential complement-directed therapeutic targets for impacting neurological disorders. Inhibition of complement-mediated synaptic pruning has been proposed using either anti C1q antibodies or small molecule inhibitors of C1r or C1s. Fusion of complement control protein domains, peptide inhibitors of C3, and modulation of endogenous inhibitors or of amplification events are all being explored and recently reviewed [271, 272]. However, systemic and constant inhibition of these key proteins has some concerns that must be balanced with any benefit to be achieved. Data mentioned above support the hypothesis that in addition to complement-mediated synaptic pruning, the activation of the complement pathway by specific misfolded proteins, cellular debris, or other neurological pathologies contributes to a local C5a-dependent polarization of microglia, leading to accelerating inflammation and neuronal dysfunction. The fact that PMX205 was effective when delivered during the period of time that Aß plaques are normally accumulating in mouse models [94] suggests that inhibition of complement-induced inflammation might substantially slow the “snowballing” cascade of cognitive decline in AD, even after the initial stages of impairment (such as mild cognitive impairment or early-stage AD) are diagnosed. Such a scenario is likely in other neurodegenerative disorders as noted in preclinical studies above. Whether C5aR1 antagonism will influence synaptic pruning and/or clearance in general and, if or if not, what the impact on cognitive or neurologic function in humans will be, remains to be seen.

Importantly, for translation of these studies to the clinic, it is critical that the targeted intervention does not have insurmountable adverse effects in humans. With regard to targeting C5aR1 signaling, there are optimistic data from clinical experience with an anti-C5 antibody. Eculizumab is an FDA-approved anti-C5 monoclonal antibody that binds C5 preventing its cleavage by C5 convertase, and thus obviating the generation of the C5b-9 membrane attack complex as well as C5a. While it has been used in humans for relatively rare specific autoimmune disorders [273] for nearly two decades, its use for additional indications is currently being tested. Thus far, a lack of C5a generation and thus C5aR1 signaling has not been harmful for extended clinical use in adults. Furthermore, IFX-1, an anti-C5a monoclonal antibody, was shown to be well tolerated in humans [274]. CCX168 (Avacopan), a small molecule C5aR1 antagonist [275], completed a promising Phase 3 clinical trial for ANCA-associated vasculitis (NCT02994927), with ongoing additional clinical trials for other disorders. C5aR1 antagonist PMX53 showed no detrimental toxicity in human clinical trials of refractory rheumatoid arthritis [276], and PMX205 with its greater access to the brain than PMX53 [277], is in development for clinical trials by Alsonex Pharmaceuticals for neurodegenerative diseases, including motor neuron disease and ALS. In addition to enhanced potential for brain permeability, an advantage of a selective small-molecule C5aR1 antagonist vs eculizumab for long-term treatment of diseases such as AD is that, while the antagonist blocks the C5aR1-mediated effects of C5a, the generation of C5b by the uninhibited cleavage of C5 is still able to initiate the assembly of the bacteriolytic C5b-9 complex upon infection-mediated complement activation, avoiding the need for vaccination against specific bacterial strains. Furthermore, since the activity of C5aR1 antagonists is quite specific [278–280], C5a is still able to interact with C5aR2, previously known as C5L2 [281]. While the function of C5aR2 is not completely characterized, it is believed to be a decoy nonsignaling receptor that scavenges C5a and that also has suppressive effects on other innate immune effector mechanisms such as the NLRP3 inflammasome [84]. It is important to note that in vitro, C5a contributes to neuronal apoptosis [282] and decreased cell viability [36, 85, 223], all of which are prevented by the C5aR1 antagonist, PMX53, or the genetic absence of C5aR1. Thus, C5aR1 antagonism may also provide direct neuroprotection.

## Summary

Prevalence of neurodegenerative diseases worldwide is accelerating. The number of individuals with Alzheimer’s disease alone in the USA is projected to reach 13 million by 2050 creating a great financial as well as emotional burden. With no curative or disease-modifying therapeutics available, there is currently a significant unmet medical need to develop therapies for AD. Evidence of excessive complement-mediated synapse pruning as well as the presence of complement activation products as evidence of the activation of the entire complement cascade within the brain of many neurological disorders leads to the hypothesis that modulation of specific complement components for different indications and stages of disease could serve as an effective therapeutic approach (Fig. 2). In animal models, direct and specific inhibition of the function of the C5aR1 while leaving the beneficial functions of other complement components such as C1q and C3b intact had beneficial effects on both pathology and cognitive behavior in several murine models of neurodegenerative disorders. However, different points of inhibition may be optimal for other disorders, particularly those involving a compromised blood-brain barrier (such as stroke, TBI, and MS). In addition, it is clear that therapeutics for acute neurological injury will have different considerations than that of chronic degenerative disorders. Fortunately, there is a growing arsenal of therapeutics specific for control points of the complement system in drug development and clinical trials [271]. The goal is to match the key pathologic drivers of a disease to the therapy while maintaining sufficient systemic protection and homeostasis provided by this ancient but well-honed system.

Human tissue is presented in italics

*ND* not determined

## Data Availability

Not applicable.

## Abbreviations

AD: Alzheimer’s disease
ADE: Astrocyte-derived exosomes
ALS: amyotrophic lateral sclerosis
ASO: Antisense oligonucleotide
Aβ: Amyloid beta
C3aRA: C3a receptor antagonist
C4BP: C4-binding protein
CCI: Controlled cortical impact
CLU: clusterin
CPN: Carboxypeptidase N
CR1: Complement receptor 1
CSF: Cerebral spinal fluid
CVF: Cobra venom factor
DAA: Disease-associated astrocytes
DAM: Disease-associated microglia
EAE: experimental autoimmune encephalomyelitis
GWAS: Genome-wide association study
LPS: Lipopolysaccharide
LOAD: Late-onset Alzheimer’s disease
MAC: Membrane attack complex
MASP: Mannan-binding lectin serine protease
MBL: Mannan-binding lectin
MCAO: Middle cerebral artery occlusion
MCI: Mild cognitive impairment
MS: Multiple sclerosis
PICALM: Phosphatidylinositol-binding clathrin assembly protein
SE: Status epilepticus
SOD1: Superoxide dismutase 1
SRPX2: Sushi repeat protein X2
TBI: Traumatic brain injury
TLE: Temporal lobe epilepsy
TLR: Toll-like receptors
SNP: Single-nucleotide polymorphism
Vsig4: V-set immunoglobulin in domain containing 4 CRIg
